# RNA regulation in brain function and disease 2022 (NeuroRNA): A conference report

**DOI:** 10.3389/fnmol.2023.1133209

**Published:** 2023-03-13

**Authors:** Monika Piwecka, Agnieszka Fiszer, Katarzyna Rolle, Marta Olejniczak

**Affiliations:** Institute of Bioorganic Chemistry, Polish Academy of Sciences, Poznań, Poland

**Keywords:** RNA biology, transcriptomics, RNA processing, miRNA, non-coding RNAs, neurodegenerative disorders, RNA therapeutics, brain tumors

## Abstract

Recent research integrates novel technologies and methods from the interface of RNA biology and neuroscience. This advancing integration of both fields creates new opportunities in neuroscience to deepen the understanding of gene expression programs and their regulation that underlies the cellular heterogeneity and physiology of the central nervous system. Currently, transcriptional heterogeneity can be studied in individual neural cell types in health and disease. Furthermore, there is an increasing interest in RNA technologies and their application in neurology. These aspects were discussed at an online conference that was shortly named NeuroRNA.

## Introduction

Modern molecular and cellular biology are more and more fostered by high-throughput approaches and jointly provide novel research tools to accelerate our understanding of how biological systems are built and regulated. Neuroscience is propelled by quickly advancing genomics, transcriptomics, and other -omics technologies; CRISPR-based gene-editing technology; the development of new models such as induced pluripotent stem cells (iPSCs)-derived brain organoids; high-resolution structural and functional deep brain imaging methods; among others. Interdisciplinary approaches advance the knowledge of basic regulatory processes in neural cells, re-visit their heterogeneity and connectivity, and eventually, give promise for developing better treatment strategies for patients suffering from neurological disorders.

RNA biology bestows a rich resource for neuroscience. It provides methods for studying gene expression in space and time, across different genotypes/phenotypes, and among different neural cell types. RNA biology also provides research tools for perturbing gene expression, which is used to perform basic research and is increasingly tested for biomedical translational purposes. Neural cells possess highly complex and multi-level gene expression regulation mechanisms and appear evident both for the transcriptional and post-transcriptional regulatory processes. Cell type-specific subsets of genes are being recognized to be regulated and coordinated at the RNA level, e.g., in response to activity inputs, injuries, or neuroinflammation. Transcriptional heterogeneity of neural cells within individual populations (e.g., interneurons) is studied in depth with single-cell RNA sequencing (scRNA-seq) and reveals unprecedented intra-populational differences (Romanov et al., 2017; Mayer et al., 2018; Muñoz-Manchado et al., 2018; Polioudakis et al., 2019; La Manno et al., 2021; Perez et al., 2021; Kamath et al., 2022), which are revolutionizing traditional classifications of neural cells based on morphology and physiology (e.g., neurotransmitter release). RNA processing is being steadily more integrated into nuclear, axonal, and synaptic signaling networks in neurons (Wong et al., 2017; Holt et al., 2019). RNA localization and subcellular modes of action of different RNA-binding proteins as well as localized translation are still under intensive investigation not only in neurons but also in other neural cell types in health and disease (Nussbacher et al., 2019; Koester and Dougherty, 2022). Moreover, aberrant RNA processing has been associated with many neurological dysfunctions. Neuropathologies, as well as neuromuscular disorders, are scrutinized, e.g., for targeting RNA splicing as a novel treatment option. RNA represents not only a target but may also serve as a source of biomarkers (Drew, 2019; Fyfe, 2021). Importantly, RNA technologies are applied for designing RNA-based therapeutics; e.g., RNA interference (RNAi) and CRISPR/Cas13 technologies are tested for providing treatment interventions in rare neurological disorders (Duarte and Déglon, 2020; Morelli et al., 2022). In fact, contemplation about how contemporary RNA biology aligns with neuroscience may come in many flavors and fascinating directions.

**Figure 1:**
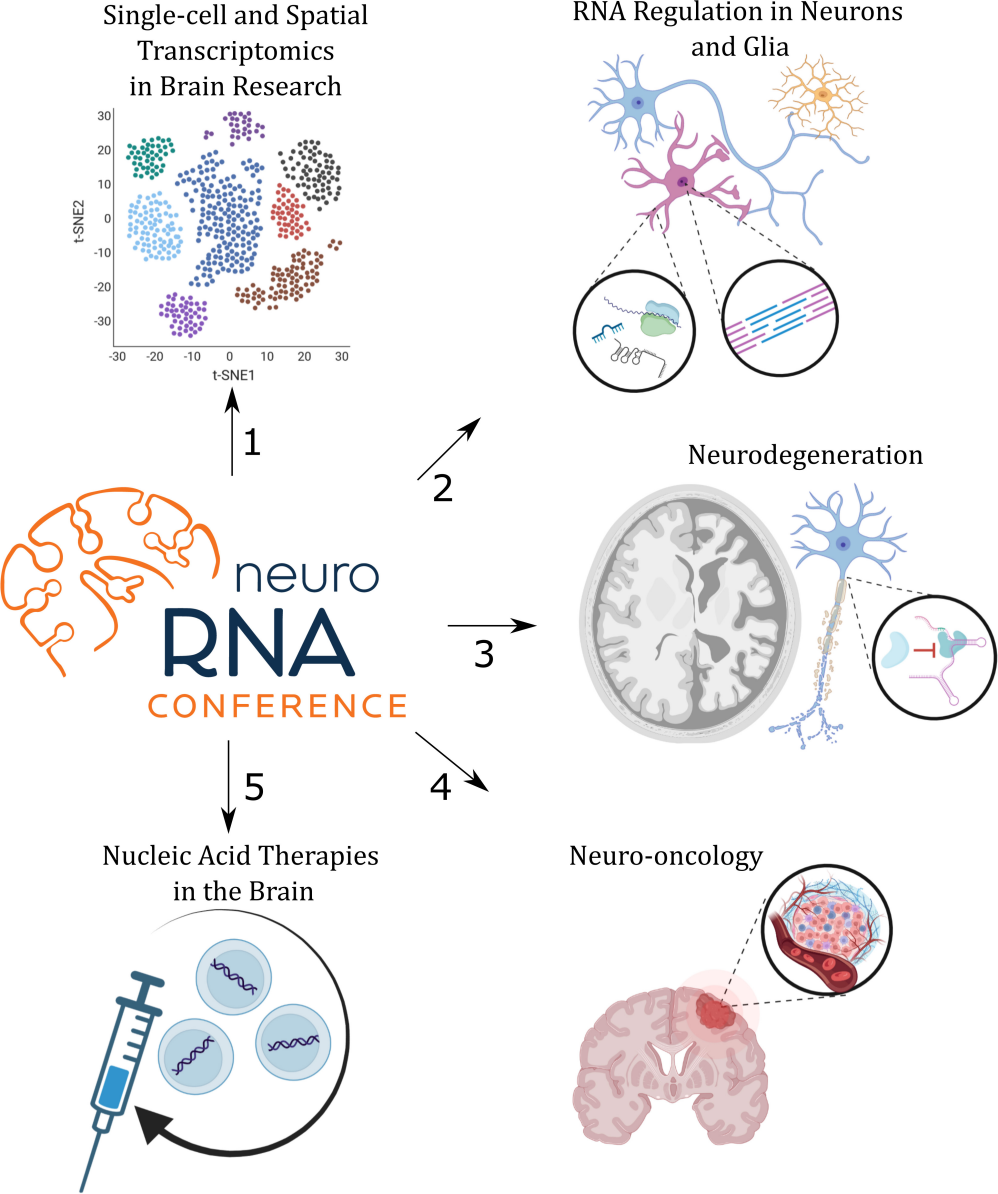
The program of the NeuroRNA 2022 Conference was divided into five sessions (1–5) that focused on specific topics as indicated.

A virtual conference *RNA Regulation in Brain Function and Disease* was organized to discuss the state-of-the-art research at the interface of RNA biology and neuroscience, with the focus on five themes arranged in five conference sessions (Figure 1); each session is briefly summarized in the following paragraphs. The organizing committee aimed to integrate and discuss novel insights into the central nervous system (CNS) and its dysfunctions from the systems biology perspective to finer molecular and cellular scales. To do that, we brought together a panel of experts in the field and young principal investigators who shared with attendees' novel results, views, expertise, and insights into cutting-edge methods. The NeuroRNA Conference (https://neurorna2022.com/) took place from 28 to 30 September 2022. There were over 200 participants from 17 countries, 17 invited talks, 13 selected talks, and 33 poster presentations.

### Living in an exciting era of single-cell transcriptomics: Impressions on the keynote lecture

The opening keynote lecture entitled “*Cellular architecture of the human brain*” was given by *Sten Linnarsson* from the Karolinska Institute, Stockholm, Sweden. ScRNA-seq technologies are gaining momentum in neuroscience, and *Sten Linnarsson* has contributed to both the inception and development of these technologies. Importantly, in the Linnarsson lab, scRNA-seq has been continuously used to uncover the complexity of the mammalian brain. During the conference, the participants had a chance to hear about two new studies, which, shortly after the meeting, became publicly available as preprints (Braun et al., 2022; Siletti et al., 2022). The first study's aim has been to map the cellular composition of the adult human brain with single-nucleus transcriptomics to obtain a highly resolved molecular atlas. The project has been run in collaboration with the Allen Institute for Brain Science within the BRAIN Initiative Cell Census Network (BICCN) and sampled over 100 anatomically distinct locations from three adult brains (each run in technical duplicate), to perform analysis of an impressive number of >3.3 million of high-quality cells. That large-scale effort revealed a great transcriptomic diversity across the adult human brain cell types and subtypes that have been defined on the more general level by 31 super-clusters. Super-clusters represent the main categories of cells and reflected the anatomical distribution and developmental origin of cells. Some neuronal super-clusters corresponded to cortical layers and projection patterns of pyramidal neurons, whereas others clearly corresponded to the neuron's developmental origin (MGE and CGE interneuron clusters corresponding to medial and caudal ganglionic eminences-origin, respectively) or distinct non-neuronal populations. Cells within super-clusters have been assigned to >3,300 individual clusters that correspond to specific individual cell types as defined based on their transcriptomes. One surprising super-cluster named “splatter” neurons was mainly found in subcortical brain regions and was composed of 92 different clusters. These cells seemed very heterogeneous when it comes to neurotransmitter identity and yet formed a complex ‘family' of transcriptionally related neurons. The breadth and wealth of the data enabled further inspection including sub-clustering into plausible cell subtypes. The data also give additional insight into the spatial region-specific distribution of glial cells, e.g., mature oligodendrocytes and their progenitors (OPCs), and astrocytes. Switching to the cell atlas of the human developing brain, >1.6 million cells were analyzed from 26 embryos (Braun et al., 2022). The study disentangles region specificity of different cell types, including radial glia, neuroblasts, and glioblasts. That dataset was used, among others, to re-visit corticogenesis. Altogether, both studies represent the most comprehensive resources available to date of individual cell transcriptomes derived from the adult and developing human brain. These resources will benefit future comparative studies, e.g., focusing on neuropathologies.

### Single-cell and spatial transcriptomics in brain research

Single-cell technologies are becoming very popular these days, and the rapid evolution of scRNA-seq methods has led to manifold discoveries over a short time. Numerous scRNA-seq studies have been reported to date, including resources of single-cell transcriptomes from different brain regions and developmental time points from human, primate, and rodent samples, from both homeostatic and multiple pathological conditions, form cell type-enriched samples (e.g., FACS-sorted), or obtained from unsorted single-cell or single-nuclei suspensions. In turn, spatial transcriptomic methods bypass tissue dissociation and retain spatial information. This allows for the assessment of gene expression across thousands of cells within the context of the entire structural organization of tissue in normal physiology or under perturbation. In the first scientific session of the conference “*Single-cell and Spatial Transcriptomics in Brain Research*,” the state-of-the-art and future perspectives of the single-cell field in the context of neuroscience have been discussed.

Single-cell RNA sequencing (scRNA-seq) has become revolutionary in expanding our understanding of complex biological systems such as brain tumors and their microenvironment. *Bozena Kaminska* (Nencki Institute of Experimental Biology, Polish Academy of Sciences, Warsaw, Poland) and her lab apply scRNA-seq and most recently spatial transcriptomics in order to study gliomas, with the main focus on glioma-associated brain macrophages and microglia and their heterogeneity in mouse glioma models. They observed a subpopulation of so-called glioma-activated microglia and were able to detect marker genes that clearly distinguish these cells from homeostatic microglia, resident macrophages, and macrophages that infiltrate the tumor. However, both macrophages and microglia shared the signatures of signaling-associated genes, and at this level, these different myeloid cells were indistinguishable. Further application of CITE-seq [Cellular Indexing of Transcriptomes and Epitopes by sequencing (Stoeckius et al., 2017)] that takes the advantage of transcriptomics and a panel of surface protein markers helped to dissect the differences between mRNA and protein abundance for a panel of genes with the single-cell resolution. The presented results were in line with the notion that monocytes/myeloid cells in the tumor microenvironment are often immunosuppressive (Munn and Bronte, 2016), additionally showing that the phenotypes can transit from proinflammatory monocytes to tumor-supportive macrophages in the brain tumor microenvironment. Interestingly, the early results show that the frequency of the switch between monocytes and tumor-associated macrophages tends to be sex-dependent or sex-biased.

A strong trend in neuroscience, and also other disciplines, has been shifting toward determining high-throughput information from multiple experimental modalities within individual cells. *Ozgun's Gokce* (Ludwig Maximilian University, Munich, Germany) illustrated how spatial transcriptomics may be combined with the measurement of morphological features afforded by electron microscopy. Gokce lab studies dementia and focuses on the identification of the cellular responses associated with the loss of white matter (WM) volume, which is one of the hallmarks of a dementing brain (Bethlehem et al., 2022). White and gray matter aged rodent brains were dissected with scRNA-seq which allowed the identification of white matter-associated microglia (WAMs) along with three subpopulations of homeostatic and activated microglia (Safaiyan et al., 2021). Careful examination of microglia transcriptomes in WM of aged brain and cross-comparison with known signatures of microglia showed that WAMs share some features with disease-associated microglia (DAM), especially when it comes to activation of genes implicated in phagocytic activity and lipid metabolism. Another observation is that there is a great loss of oligodendrocytes in WM of 24-month-old mice (Kaya et al., 2022) and that these cells are also changing their phenotypes, e.g., a sub-population of “interferon response” oligodendrocytes was noticed in the white matter of aged brains. A new development from Gökçe lab, spatial transcriptomics-correlated electron microscopy (Androvic et al., 2022) combines multiplex error-robust FISH [MERFISH (Chen et al., 2015)] information with EM. It was applied to study the spatial context of glial cells in WM. In the proof-of-principle study, it enabled to provide a link between the morphology of “foamy” microglia (featuring lipid droplets inside the cell) and a tiny population of interferon-response microglia with their transcriptional signatures and proximity to T-cells (Androvic et al., 2022). The application of single-cell technologies and spatially resolved molecular approaches gives the opportunity to provide unprecedented depth into many physiological states of glial cells in a high-throughput data-driven fashion. It can also provide new clues to the relevance of the co-existence of different cell types/subtypes in spatial proximity. How about single-cell chromatin profiling? *Marek Bartosovic* (Karolinska Institute, Stockholm, Sweden) switched gears to “*Multimodal profiling of the epigenome at single-cell resolution”*, particularly focusing on the application of CUT&Tag in single nuclei obtained from juvenile (P15, P25) mouse brain. That setup enabled the study of multiple histone marks and transcription factors Olig2 and Rad21 at single-cell resolution and cluster cells according to their chromatin profile (Bartosovic et al., 2021). These datasets allowed also the prediction of cell-type-specific promoter-enhancer interactions. In addition to the measurement of open chromatin regions, now it is possible to capture multimodal epigenetic marks in single cells. To achieve that, the new nanobody-Tn5 fusions are being used to target and barcode two distinct active and repressive histone marks (Bartosovic and Castelo-Branco, 2022). This new method termed nano-CUT&Tag is of much better sensitivity as compared with scCUT&Tag and allows for the discrimination of more cell types/states together with the possibility to infer chromatin velocity to predict differentiation trajectories of distinct cell types. Dissection of the oligodendrocyte population with nano-CUT&Tag revealed the presence of two sequential waves of H3K27me3 repression at distinct gene groups during the lineage differentiation. A recent introduction of a cutting-edge method combining spatial co-profiling of gene expression (RNA) and spatial chromatin profiling was also mentioned (Deng et al., 2022).

*Agnieszka Rybak-Wolf* (Berlin Institute for Medical Systems Biology, Berlin, Germany) presented an article on herpes simplex virus (HSV)-driven encephalitis. HSV-1 infection was evoked in human brain organoids and studied at the molecular and physiological levels. The study recapitulated known features of HSV-1 infection (such as diminished synaptic gene expression and decreased synaptic firing) and delivered novel insights into perturbed gene expression due to infection, e.g., activation of antisense transcription and global increase in the length in poly(A) tails in mRNA (Rybak-Wolf et al., 2021). Single-cell RNA-seq in HSV-1-infected organoids revealed an overall changed cellular composition of organoids upon infection and a few clusters of cells (aka cell types) that were more susceptible to infection (“highly infected” cells). In turn, acyclovir (ACV)-treated organoids featured low virus load, yet their transcriptomes were not back to normal “pre-infection” profiles. In particular, the TNF-α signaling pathway was still upregulated post-ACV treatment in certain cell types. Attenuation of inflammatory response with anti-inflammatory drug rescued some infection effects in organoids, such as recovered neuroepithelial integrity. The presented work provided robust evidence for the relevance of brain organoids as a model system for studies on brain infections and the usefulness of scRNA-seq in tracing therapeutic outcomes of new therapies and drug re-purposing. Another study that took advantage of brain organoids as a model system was presented in a short talk by *Ivano Legnini* (Berlin Institute for Medical Systems Biology, Berlin, Germany) who showed the validity of the new optogenetic method for programmable silencing or activation of target genes. Light-inducible CRISPR/Cas9 construct and gRNAs were used to target and activate transcription from *Sonic Hedgehog* (SHH) promoter in a proof-of-concept experiment (Legnini et al., 2022). Importantly, such a system allows the programming of gene expression only in intended cells. By light-controlled activation of a morphogen (such as *SHH)* in 3D brain organoids, one can provide a stimulus to develop an organoid with a certain spatial patterning mimicking *in vivo* situation, i.e., developing neural tube-like in case of *SHH* activation. The analysis of optogenetically patterned brain organoids was performed both with single-cell and spatial transcriptomic methods.

The following short talks in this session provided a transition to the second scientific session about the mechanisms of gene expression regulation in brain cell types. *Franz Ake* (Bellvitge Institute for Biomedical Research IDIBELL, Barcelona, Spain) discussed the insights into the challenges and importance of detecting alternative polyadenylation (APA) in individual cells. Is differential APA attributed to neurological conditions and can that be investigated from the single-cell transcriptomic datasets generated with currently available platforms? To tackle that question, Mireya Plass lab applies single-cell isoform quantification in iPSC-derived neurons from patients with Alzheimer's disease (AD) and isogenic controls using an in-house developed workflow. Preliminary results show a subset of genes that switch mRNA isoforms in AD pathology as compared with controls and that it occurs in a cell type-specific manner in heterogeneous neuronal cells differentiated from iPSCs. In turn, *Rotem Ben Tov Perry* from the Weizmann Institute of Science (Rehovot, Israel) presented a study about long-non-coding RNA (lncRNA) called *Silc1*. *Silc1* RNA has been previously shown to be important for cis-activation of the transcription factor *Sox11* during neuroregeneration in the peripheral nervous system (Perry et al., 2018), and now its role has been dissected in the CNS. In the presented unpublished results [now available as a preprint (Perry et al., 2022)], the authors highlighted transcriptional induction of *Silc1* upon stimulation in the hippocampus and evidenced its role in neural plasticity and memory formation in spatial learning.

### RNA regulation in neurons and glia

In the second scientific session, a few timely subjects regarding RNA regulation in neural cells have been discussed. RNA splicing, the choice of transcription start site (TSS), and poly(A) site have fundamental roles in mRNA maturation, stability, and localization, and all of these ultimately influence the gene expression programs, oftentimes in a cell type-specific manner. *Hagen Tilgner* (Weill Cornell Medicine, New York, USA) provided a comprehensive overview of the current understanding of alternative splicing and mRNA isoform usage in the brain that is now being dissected with single-cell resolution. The application of single-cell isoform RNA sequencing (Gupta et al., 2018) highlighted a cell type specificity at the isoform level for a subset of mRNAs as well as brain-region specificity of different isoforms (Joglekar et al., 2021). That phenomenon has functional consequences on the occurrence of different protein isoforms and may impact distinct subcellular localization patterns of either RNA or the protein. Tilgner lab is moving forward with single-nuclei RNA sequencing (snRNA-seq) and isoform identification that enables analysis of frozen tissue material (i.e., the majority of clinical samples). The key methodological development in SnISOr-seq (single-nuclei isoform RNA sequencing) is the addition of two steps in cDNA library preparation: an asymmetric PCR to amplify barcoded cDNA and an enrichment step using exon-targeting probes to filter out purely intronic molecules (Hardwick et al., 2022). As alternative exon inclusion/skipping event is relatively common in the brain and cell type-specific phenomenon, more interestingly, there is cell type-specific coordination of exons, TSS, and poly(A) patterns that can be noticed from snRNA-seq datasets (Hardwick et al., 2022).

*Joseph Dougherty* (Washington University School of Medicine, St. Louis, USA) talked about alternative translation and local translation in glia, and it was a very valuable contribution to discussions regarding the regulation of alternative mRNA isoforms in brain cells. Local translation in neurons is nowadays an intensively studied and well-recognized phenomenon (Holt, Martin, and Schuman 2019). The recognition of local translation in other neural cells, such as astroglia, has been emerging recently. From the Dougherty lab's earlier work, we have learned that translation exists in distal astrocytic processes (Sakers et al., 2017), and that line of research has been extended to microglia (Vasek et al., 2021). Microglia have been shown to perform local protein synthesis in peripheral microglial processes, particularly at the perisynaptic and phagocytic structures. In fact, a specific subset of mRNAs is being translated at the peripheral process of microglia as shown by profiling of the ribosome-bound mRNAs from processes and the enrichment of phagocytosis-related transcripts. From the other angle, it was evidenced that translation is required for phagocytic cup formation and sufficient to enable phagocytosis in microglia processes that were severed from the cell somas. Switching to alternative translation, that process depends on differential usage of translation initiation sites on one transcript. Similarly to alternative splicing, alternative translation diversifies proteome and allows for greater biological complexity. The Dougherty lab showed that the choice of translation initiation site might be activity-dependent in neurons (Sapkota et al., 2019). Another interesting observation coming from translating ribosome affinity purification to ribosome footprinting is a cell type-specific mechanism for translational stop codon readthrough in the mouse brain (Sapkota et al., 2019).

In recent years, the community has made substantial progress toward the detection and profiling of non-coding RNAs (ncRNAs) in the mammalian brain and multiple pathological conditions, e.g., in the past 10 years, numerous studies have informed about changes in microRNA (miRNA) levels in the different brain regions of patients suffering from neurological disorders and in neural tissues from animal models of respective conditions. Functions and mechanism of actions of newly annotated ncRNAs in the majority of cases remain unclear, albeit there is still a growing interest in research focusing on disentangling ncRNA impact on gene expression, as some of them have been evidenced to possess important regulatory functions. Functional studies in animal models provide evidence that certain miRNAs influence pathogenesis, e.g., genetic deletion of miR-128, a crucial regulator of neuronal excitability, leads to fatal epilepsy in mice (Tan et al., 2013). During the conference, *Jeroen Pasterkamp* from the University Medical Center Utrecht introduced his lab's work on ncRNAs in neurological disease, namely, miRNAs and circular RNAs (circRNAs) and their association with temporal lobe epilepsy (TLE). miRNAs and miRNA-binding proteins Argonautes are mainly localizing to the cytoplasm in cells, yet there are exceptions from that commonly accepted rule (Leung, 2015). What are the function and the mechanism beyond nuclear miRNAs, still remains unclear. Abnormal nuclear localization of a subset of miRNA in TLE was observed and now gets dissected to provide new knowledge about the mechanism of nuclear miRNA action. The recent insight into circRNAs in neurons and their newly evidenced role in the regulation of dendritic spine morphology was also highlighted (Gomes-Duarte et al., 2022). The subject of ncRNA in brain research was continued with the selected talks. *Mollie K. Meffert* (The Johns Hopkins University School of Medicine, Baltimore, USA) presented her group's ongoing work on the abundant let-7 family of miRNAs and its molecular targets and function in the brain. *Fmr1* KO mice have been used in the study, and they showed an activity-dependent reduction of let-7 levels in behavioral studies. *Orna Issler* (Icahn School of Medicine at Mount Sinai, New York, USA) introduced the topic of sex-specific lncRNAs in the context of depression, and more generally — mood. A previous study highlighted that a substantial part of the differentially expressed genes in the brains of depressed humans (~1/3) belong to lncRNA-encoding genes and that these transcripts display region- and sex-specific patterns of the regulation (Issler et al., 2020). Her current project focused on one particular lncRNA called FEDORA, which was found to be positively correlated with depression susceptibility in women (Issler et al., 2021). Last but not the least, a new insight into circRNA Cdr1as association with brain homeostasis over daily light–dark (LD) cycles was introduced by *Andranik Ivanov* (Berlin Institute of Health, Berlin, Germany). Cdr1as is one of the best studied to date brain-enriched circRNAs. It has the unique property of possessing multiple binding sites for miRNA, in mice >120 binding sites for miR-7 and one binding site for miR-671. Cdr1as was previously shown to be involved in the regulation of miR-7 and its mRNA targets in excitatory neurons in multiple regions of the mouse brain. *Cdr1as* KO mice were identified with behavioral abnormalities, namely, sensorimotor gating phenotype that is associated with neuropsychiatric disorders and dampened neural activity in *in vitro* autaptic neuronal cultures (Piwecka et al., 2017). However, the study by Ivanov et al., brings another layer of complexity to *Cdr1as* functions in neurons and circRNA stability, as it clearly implies that Cdr1as undergoes dynamic turn-over in the suprachiasmatic nucleus over the LD cycles (Ivanov et al., 2022).

### RNA in brain pathology: Neurodegeneration

This session was focused on recent findings and challenges in studying RNA molecules in neurodegenerative disorders. Mutant forms of RNAs can trigger disruptions in molecular pathways that specifically affect neurons, which further contribute to neurodegeneration. Global deregulation of the transcriptome is a common observation in the diseased brain and its investigation with RNA-seq helps to understand the pathology at the molecular level. The presentations in this session covered some of these aspects in Parkinson's disease (PD) and Alzheimer's disease (AD), and repeat expansion diseases such as Huntington's disease (HD).

*Gracjan Michlewski* (International Institute of Molecular and Cell Biology, Warsaw, Poland) presented insights into specific RNA–protein interaction networks. RNA-binding protein HuR — pri-miR-7 — α-synuclein (*SNCA*) mRNA has been investigated in the context of PD. This regulatory network provides an option for therapeutic intervention in PD, as increased levels and aggregation of α-synuclein are a hallmark of this disease, and miR-7 biogenesis *via* HuR might be targeted to impact *SNCA* expression (Poewe et al., 2017). The RNA Pull-Down COnfocal NAnoscanning (RP-CONA) method was used to identify compounds that may disrupt pri-miR-7–HuR interaction (Zhu et al., 2021) and that line of research is further developed in Michlewski lab.

*Evgenia Salta* (Netherlands Institute for Neuroscience, Amsterdam, the Netherlands) referred to the cellular and molecular complexity of AD pathology, including complex miRNA regulation and challenges in the use of miRNAs as therapeutics (Walgrave et al., 2021b). The focus of current research in the Salta lab is on revealing coding and non-coding regulators of adult hippocampal neurogenesis in AD. Based on previous results regarding miR-132 downregulation in AD (Walgrave et al., 2021a) and its role in neurogenesis, miR-132 has been further studied in the context of impaired neurogenesis in AD. More details of this process are expected to emerge from ongoing scRNA-seq of AD patients' brains, specifically regions containing neurogenic niches. Interestingly, samples from non-demented patients with AD are included in these analyses.

*Pawel Switonski* (Institute of Bioorganic Chemistry, Polish Academy of Sciences, Poznan, Poland) is studying neuronal vulnerability to degeneration in the spinocerebellar ataxia type 7 (SCA7), one of the CAG repeat expansion diseases. Purkinje cells (PCs) represent the main cell type that undergoes degeneration in SCA7. They constitute approximately 2% of cells in the cerebellum and are one of the biggest and most highly structured neurons in the adult brain, thus it is impossible to isolate them without losing the integrity of neuronal processes. In the presented study, the author described the challenges and eventually the method on how to specifically enrich single nuclei of PCs from the mouse cerebellum. The nuclei have been subjected to snRNA-seq. Based on the established PC isolation method, further studies are being conducted to unravel PC-specific molecular mechanisms leading to neuronal death (Switonski et al., 2021).

In the two following short talks, insights into RNA biology in the context of the other repeat expansion diseases have been presented. First, *Katarzyna Tutak* (Institute of Molecular Biology and Biotechnology, Adam Mickiewicz University, Poznan, Poland) presented details of the mechanism of repeat-associated non-AUG (RAN) translation occurring at repeated expanded tracts in fragile X-associated tremor/ataxia syndrome (FXTAS) (Baud et al., 2022). RAN translation results in the production of toxic glycine-rich protein (polyG) derived from expanded CGG repeats (Guo et al., 2022). Attempts to identify the modifiers of this process have been discussed, as the new knowledge may open up novel possibilities for designing of therapeutic strategy for FXTAS. In another presentation, *Pawel Joachimiak* (Institute of Bioorganic Chemistry, Polish Academy of Sciences, Poznan, Poland) referred to the allele-specific analysis of transcript levels for selected polyglutamine (polyQ) diseases. Patients suffering from polyQ diseases usually possess both normal and mutant alleles of the affected gene (Bunting et al., 2022). Investigating mRNAs from genes implicated either in HD or spinocerebellar ataxia type 3 (SCA3) provides information about disproportions in allelic expression, as well as changes in expression levels during neuronal differentiation. This is a starting point for detailed analyses of mutant mRNA-specific processes (Ciesiolka et al., 2021; Joachimiak et al., 2022), which are important for a precise description of molecular mechanisms underlying polyQ diseases. In the last short talk in this session, *Sambhavi Puri* (Boston University School of Medicine, Boston, USA) shared the results on circRNAs deregulation in AD. Altogether, 48 circRNAs have been identified to be associated with AD conditions based on RNA-seq data generated from the hippocampus and the cortex of patients' brains. Moreover, circRNA expression was shown to differ by dementia subtype. The importance of impaired circRNA expression patterns and their association with pathogenesis are still emerging topics in the AD field.

### RNA in brain pathology: Neuro-oncology

Brain tumors are complex diseases resulting from the disruption of key cellular pathways, including those regulating cell survival and division. Genetic mutations, perturbed RNA profiles, and epigenetic alterations contribute to tumorigenesis. It is already known that all these changes are heterogeneous on a cell type basis within an individual tumor and between patients, which is increasingly appreciated as a determinant of treatment failure and disease recurrence in the case of brain tumors. During this conference session, we discussed the intratumor heterogeneity, new mechanisms of regulatory interactions between ncRNAs, and the possibilities for the identification of new RNA and epigenetics-oriented therapeutics and diagnostic approaches.

*Itay Tirosh* (Weizmann Institute of Science, Rehovot, Israel) presented a single-cell transcriptomic analysis of glioblastoma samples that recapitulated the high intratumoral heterogeneity of the most aggressive brain tumor. The presented scRNA-seq data showed that glioblastoma cells exist in four main cellular states. The authors found that these states might be affected by the tumor microenvironment and exhibit plasticity, i.e., multiple possible transitions between states can be observed per transcriptome level. The relative frequency of cells in each state also varies between glioblastoma samples, and it is influenced by copy number amplifications of the *CDK4, EGFR*, and *PDGFRA* loci and by mutations in the *NF1* locus (Neftel et al., 2019). Further analyses of pan-cancer data regarding intratumoral heterogeneity of the different cancer types revealed that transcriptomic profiles appear to be inherently variable when it comes to gene expression related to oncogenic signaling, proliferation, complement/immune response, and hypoxia (Gavish et al., 2021).

The presence of glioblastoma stem-like cells (GSCs) within the tumor mass, hypoxic microenvironment, and poor infiltration of immune cells to the core of the tumor is perceived to be the other reasons why anti-tumor therapies fail to be efficient. *Agnieszka Bronisz* (Mossakowski Medical Research Institute, Polish Academy of Sciences, Warsaw, Poland) presented an interesting therapeutic approach designed to target GSCs with the oncolytic herpes virus (oHSV)-based immunotherapy. It was illustrated in the talk that oHSV infection alone may activate the host antitumor immune system by triggering “immunosecretion”, i.e., the release of antigens and cytokines that stimulate the immune response. The presented data showed that upon infection with oHSV and the transcriptome- and secretome-associated proteins are regulated in GSC. The observed changes have been linked to T-cell-mediated cytotoxicity and B-cell-dependent immune response memory. On the other hand, detailed downstream analyses identified the presence of stress-resistant and oHSV-resistant GSCs located in the hypoxic niche. Transcriptome-wide data revealed that the tumor microenvironment shapes two distinguishing characteristics of GSCs: increased cell-to-cell communication with immune cells and metabolic shift toward hypoxic adaptation, both with signatures predictive of glioblastoma patent survival. One of the antisense lncRNAs has been identified as a sensor of response and adaptation of GSCs to hypoxia. Further analyses pointed out that it might be a promising therapeutic target for glioblastoma therapy, especially in combination with oncolytic virus immunotherapy.

The relevance of ncRNAs for tumorigenesis, tumor progression, and relapse was commented on in *Anna's Krichevsky* (Brigham and Women's Hospital and Harvard Medical School, Boston, USA) talk (Brigham and Women's Hospital and Harvard Medical School, HMS Initiative in RNA Medicine, Boston, USA). In particular, the role of the network is composed of a few distinct ncRNAs including a miRNA, enhancer-associated RNA, promoter-associated RNA, and snRNA. The central part of that network is miR-10b, a miRNA that remains silent in the brain cortex and becomes upregulated in gliomas where it is known to serve a tumor-promoting role. The two mentioned lncRNAs are involved in the chromatin folding and concordant regulation of miR-10b and other co-transcribed genes. The network remains inactive in homeostatic astrocytes and becomes activated during neoplastic transformation. In addition, Krichevsky's group evidenced that direct binding between miR-10b and U6 has a strong impact on downstream U6 interaction with splicing factors SART3 and PRPF (El Fatimy et al., 2022). These results shed new light on the nuclear function of one of the major cancer-associated miRNAs.

*Zaneta Zarebska* (Institute of Bioorganic Chemistry, Polish Academy of Sciences, Poznan, Poland) presented results on circRNAs and their role in gliomagenesis and GBM progression. RNA-seq results obtained from GBM samples revealed the differential expression patterns within the tumor samples compared with the normal brain. Specific circRNAs have been found to correlate with the GBM molecular subtypes, which is another attempt for providing better stratification that may underlie the future personalized treatment of patients with GBM.

*Katarzyna Leszczynska* (Nencki Institute of Experimental Biology, Warsaw, Poland) shifted the attention to the pediatric high-grade gliomas (pHGG) and one of the recognized mutations in histone H3 that is specific for these tumors: H3F3A variant. Chromatin-modifying agents, including histone deacetylase (HDAC) inhibitors, have been identified as promising candidate therapeutics against pHGG. The treatments of HDAC inhibitors provided also new directions in targeting H3K27M-expressing cells. However, the precise correspondence between the efficiency and chromatin response of that treatment is still not completely understood. The presented data focused on the chromatin alterations induced by HDAC inhibitors and the role of histone variants in response to these therapies. With the multiple cellular models expressing the H3K27M histone variant, several new drugs with sub-micromolar efficacy in killing H3K27M-expressing cells were identified. The results pointed out that direct analysis of the chromatin landscape, and particularly the expression of histone variants in pHGG, and provide new insights into chromatin response to specific epigenetic treatments.

### Nucleic acid therapies in the brain

Many nucleic acid-based therapeutics, mainly antisense oligonucleotides (ASOs), have recently entered the clinical trial phase. This progress is particularly evident in the field of neurodegenerative diseases, such as HD, where the strategies for lowering the level of mutant protein with ASO or RNAi appear to be very promising. Unfortunately, despite many years of research and the positive results of preclinical studies, some clinical trials had to be terminated prematurely due to ASO ineffectiveness or unexpected side effects. The main problem is still the effective and minimally invasive delivery of the therapeutic to the brain and its specificity. One line of research is the development of new gene therapy constructs and viral vectors for the targeted delivery of therapeutic molecules such as RNAi triggers or CRISPR-Cas system components. Our speakers raised all these important issues in their speeches.

*Leontien van der Bent* (uniQure biopharma B.V., Amsterdam, the Netherlands) demonstrated recent advances in the development of miRNA-based gene therapies for the treatment of various CNS disorders, including HD, temporal lobe epilepsy, amyotrophic lateral sclerosis, synucleinopathies, and AD. MiQure technology relies on vector-based RNAi triggers, also known as artificial miRNAs (Kotowska-Zimmer et al., 2021), to specifically knockdown target RNAs. The advantage of this approach, as compared with ASO-based technology, is the possibility of obtaining a long-term therapeutic effect after a single administration of the viral vector. It is worth mentioning that uniQure is currently conducting Phase I–II clinical trials of the first AAV gene therapy for HD in which rAAV5-miHTT is delivered directly to the brain by MRI-guided stereotactic infusion (https://clinicaltrials.gov/ct2/show/NCT04120493). This stage of research was preceded by numerous preclinical studies in rodents, mini pigs, and non-human primate models, which demonstrated the effectiveness and safety of the approach (Spronck et al., 2021).

Undoubtedly, the last decade was the time of genome editing technology, which gave great hope in the context of gene therapy. Still, the biggest problem is the safety of the technology. *Nicole Deglon* (Neuroscience Research Center and Department of Clinical Neuroscience, Hospital and University of Lausanne, Switzerland), an expert in the field of viral gene transfer, presented her research on the development of the second-generation AAV-KamiCas9 self-activating system for the efficient and safe editing of CNS disease genes. Transient expression of the Cas9 protein decreases the risk of off-target effects, while optimized AAV vectors ensure efficient transduction of specific brain circuits affected in neurodegenerative disorders (Merienne et al., 2017).

Another class of therapeutics showing a wide spectrum of applications is ASOs. The therapeutic potential of short antisense oligonucleotide steric blockers and small compounds targeting expanded CGG repeats in FMR1 5'UTR was demonstrated by *Krzysztof Sobczak* (Institute of Molecular Biology and Biotechnology, Adam Mickiewicz University, Poznan, Poland). CGG repeat expansion reaching 55–200 triplets leads to the development of FXTAS syndrome. The toxicity of the RNA containing expanded CGG repeats results in the sequestration of nuclear proteins involved in RNA metabolism, the initiation of non-canonical translation of the polyglycine-containing protein which forms nuclear insoluble inclusion, and the formation of the R-loop structure. ASO LNA designed by the Sobczak group is specific to bind and disrupt the RNA structure formed by CGG repeats during transcription, thereby abolishing all these toxic effects. These ASOs have been shown to improve motor behavior and rescue gene expression abnormalities associated with FXTAS in a mouse model of the disease (Derbis et al., 2021). Interestingly, the authors observed non-specific upregulation of other CGG repeat transcripts; however, protein levels remained unchanged.

A careful evaluation of the effects induced by ASO was the topic presented by *Savani A. Anbalagan* (Institute of Molecular Biology and Biotechnology, Adam Mickiewicz University, Poznan, Poland) (Adam Mickiewicz University, PL). He showed that ASO targeting the splicing site of a protein-coding gene embedded in ncRNA may influence the expression and function of the ncRNA. In this study, ASO-inducing intron retention events and increased gene expression resulted in defects in axonal morphogenesis in zebrafish larvae. Without careful evaluation of this phenomenon and the application of appropriate controls, it could have been misinterpreted as an effect of the protein-coding gene.

*Lorea Blazquez* (Biodonostia Health Research Institute, San Sebastian, Spain) studies aberrant regulation of RNA processing in neurological disorders and her talk focused on frontotemporal dementia. A non-coding mutation in the *GRN* gene leads to an abnormal splicing pattern that causes *GRN* mRNA degradation and progranulin haploinsufficiency. She showed that the CRISPR-dCas13 system can not only be used to identify a target sequence in a pre-mRNA for RNA-based therapeutic strategies but also can be used as a therapeutic approach to restore the GRN open reading frame. RNA-targeting CRISPR-dCas13 has some advantages over ASO or the traditional CRISPR-Cas9 system. It can be expressed from viral vectors without the need for repeated administration and appears to be at least theoretically safer than DNA targeting Cas9. However, more research is needed to confirm its therapeutic potential.

## Conclusion

The increasing synergy between RNA biology and neuroscience brings the research in the interface of both disciplines to a new level that enables us to look into physiology with a molecular resolution and in a high-throughput manner. The first edition of the NeuroRNA conference illustrated how rapidly developing new technologies focused on RNA can enrich our understanding of the basic molecular processes in neural cells and contribute to the formulation of new research hypotheses that aim to bring us closer to unraveling the complexity of the human brain and to find cures for complex neurological conditions. In his biography, Santiago Ramón y Cajal penned “It is commonplace fact that scientific discoveries are a function of the methods used.” That notion definitely holds true when contemplating NeuroRNA 2022.

A few summary points on the presented and discussed insights that are worth highlighting as follows:

1. The repertoire of neural cell types and subtypes in the adult human brain, both neurons and glia, is much more heterogenous when considering transcriptome-based classification, i.e., >3,000 cell types discovered in the adult human brain as based on RNA single-nuclei sequencing.
2. Plasticity of certain neural cells, i.e., the possibility to acquire new states and transit between states. It is more often evidenced with scRNA-seq and trajectory inference methods (e.g., for oligodendrocytes, microglia, glioblastoma tumor cells, and glioblastoma stem cells).
3. Alternative transcript isoforms and 3'UTR usage (including APA) are commonplace in neural cells, cell type-specific, and likely context-dependent (i.e., isoform switch may occur depending on the condition). This phenomenon will be surely investigated further delivering new knowledge on how RNA isoforms influence the diversification of proteome on a cell type basis.
4. Local translation is not restricted to neurons. Other polarized and highly structured cell types feature regulated the transport of a specific subset of transcripts to perform local synthesis of specific proteins in cell peripheries, that is, evidenced by microglia and the production of phagocytosis-related factors in microglial processes, and astrocytes and local synthesis of proteins with roles in regulating synapses in astrocyte peripheral processes.
5. Subcellular localization of regulatory RNAs is one of the determinants of their versatility, e.g., miRNAs may function in a non-canonical way in the nucleus, and re-localization of regulatory RNAs might be associated with the pathology (epilepsy and tumorigenesis).
6. The landscape of non-coding RNAs which is functional in the brain is quickly progressing with many newly identified molecular roles that influence the physiology of the CNS on a cell type basis and oftentimes in a sex-based manner. Of note, a poster presentation by *Vittorio Padovano* (Sapienza University, Rome, Italy) on lncRNA in motor neurons supported by lncRNA knockout in the mouse model was awarded the best poster award at the conference.

Many alterations in the transcriptome, specific mutated transcripts, disease-specific transcript isoforms, mislocalized regulatory RNAs, etc., are carefully examined with the ultimate aim to find causative and treatable pathological targets in the CNS. A new generation of therapeutics that are more precise and specific in reaching the targets is on the horizon, and we hope that in the next incarnation of NeuroRNA, we will learn about the progress in that matter. Last but not the least, we would like to express our gratitude to the conference speakers for the inspiring talks and for sharing fresh, oftentimes unpublished results, the audience for the stimulating discussions, and the sponsors for their support.

## Author contributions

MP conceptualized the manuscript and edited the final version of the manuscript. MP, KR, AF, and MO wrote and edited the first draft of the manuscript. All authors approved the submitted version.
